# Modeling Electrostatic Force in Protein-Protein Recognition

**DOI:** 10.3389/fmolb.2019.00094

**Published:** 2019-09-25

**Authors:** H. B. Mihiri Shashikala, Arghya Chakravorty, Emil Alexov

**Affiliations:** Department of Physics, Clemson University, Clemson, SC, United States

**Keywords:** binding, electrostatics, molecular recognition, polar solvation energy, electrostatic force

## Abstract

Electrostatic interactions are important for understanding molecular interactions, since they are long-range interactions and can guide binding partners to their correct binding positions. To investigate the role of electrostatic forces in molecular recognition, we calculated electrostatic forces between binding partners separated at various distances. The investigation was done on a large set of 275 protein complexes using recently developed DelPhiForce tool and in parallel, evaluating the total electrostatic force via electrostatic association energy. To accomplish the goal, we developed a method to find an appropriate direction to move one chain of protein complex away from its bound position and then calculate the corresponding electrostatic force as a function of separation distance. It is demonstrated that at large distances between the partners, the electrostatic force (magnitude and direction) is consistent among the protocols used and the main factors contributing to it are the net charge of the partners and their interfaces. However, at short distances, where partners form specific pair-wise interactions or de-solvation penalty becomes significant, the outcome depends on the precise balance of these factors. Based on the electrostatic force profile (force as a function of distance), we group the cases into four distinctive categories, among which the most intriguing is the case termed “soft landing.” In this case, the electrostatic force at large distances is favorable assisting the partners to come together, while at short distance it opposes binding, and thus slows down the approach of the partners toward their physical binding.

## Introduction

Electrostatics plays an important role in molecular biology since it contributes to protein folding and stability (Strickler et al., 2006; McCammon, 2009; Tsai et al., 2016), protein-protein interactions (Zhang et al., 2011), ion binding (Petukh and Alexov, 2014; Petukh et al., 2015a), dimerization (Zhang et al., 2013; Campbell et al., 2014), protein-DNA/RNA interactions (Ghaemi et al., 2017), and protein-microtubule binding (Li et al., 2016a,b). It is the major component determining pKa values of ionizable groups in proteins and DNAs/RNAs (Alexov et al., 2011; Onufriev and Alexov, 2013; Wang et al., 2015; Pahari et al., 2018). Even more, electrostatics has been demonstrated to be implicated in diseases (Li et al., 2017a), since disease-causing mutations frequently alter wild type electrostatic interactions (Teng et al., 2009). Altogether, electrostatic energies and forces are essential for molecular biology (Honig and Nicholls, 1995).

As mentioned above, electrostatics is an important component of protein-protein binding and recognition (Talley et al., 2008; Zhang et al., 2011; Campbell et al., 2014; Chakavorty et al., 2016). Overall, protein-protein recognition is a complex process; involving a balance between entropy and enthalpy (Gilson and Zhou, 2007; Zhou and Gilson, 2009; Li et al., 2015). Both components undergo changes as the partners approach each other from a free state to a bound state and eventually physically bind. Among the enthalpy components, electrostatics plays dominant role at the beginning of the recognition process when partners are far away from each other. At distances larger than several water layers (water layer is typically considered to be about the average diameter of water molecule of 2.8 Å), all other energies and forces are practically negligibly small, and electrostatics is the only one guiding the recognition. As the partners approach each other, other energy terms become equally significant and the outcome of the binding depends on their balance.

Macromolecular interactions, in particular the protein-protein interactions, have been studied by many labs, both computationally and experimentally (Bagher et al., 2019; Bolla et al., 2019; Dholey et al., 2019; Ferreira et al., 2019). Some works have focused on the dynamics associated with binding, e.g., de-hydration (Ferrario and Pleiss, 2019; Gao et al., 2019; Mishra et al., 2019) and, others have worked on predicting the binding mode via various docking or homology based techniques (Hwang et al., 2017; Meyer et al., 2018; Agrawal et al., 2019; Porter et al., 2019; Wang and Dokholyan, 2019; Zhang and Sanner, 2019; Zheng et al., 2019). Of particular interest to our work are the computational investigations of Zhou (1997), Alsallaq and Zhou (2008a,b), and Pang et al. (2012) on modeling association rates of macromolecular binding. In their approach, the ligand is positioned away from the receptor such that only the electrostatics contribute to the macromolecular interactions (Qin et al., 2012). This approach was also implemented into a webserver (http://pipe.sc.fsu.edu/) (Qin et al., 2011).

In this work, we focus on the role of the electrostatic energies and electrostatic forces in protein-protein recognition. It was previously demonstrated that electrostatics contributes to molecular binding via Coulombic and polar de-solvation energies (Gilson and Zhou, 2007; Zhou and Gilson, 2009). Depending on the charge distribution, both the net charge and the charge at the binding interfaces, the Coulombic interactions may be favorable or not (Kundrotas and Alexov, 2006; Teng et al., 2009). The polar de-solvation energy is almost always unfavorable, except for the rare cases that involve binding of molecules carrying like charges (Bertonati et al., 2007). Thus, in a typical case involving favorable Coulombic interactions, the total electrostatic energy profile as function of the distance between the partners is a smooth curve with a minimum either at zero distance (bound state) or at a distance roughly corresponding to the size of a water molecule (Murray et al., 1998).

When the interacting partners, being proteins, nucleic acids, lipids, small molecules or residues, are separated by distances larger than several water layers, their interaction energy is purely electrostatic in origin. It can be modeled via the so called *screened Coulombic interactions*. For example, this can be done using DelPhi FRC module, which uses the Poisson-Boltzmann (PB) delivered electrostatic potential to find the electrostatic potential at any given point in the computational box. This is accomplished by charging one of the interacting partners and obtaining the electrostatic potential on the atoms of the other partner. Then, the screened Coulombic interaction energy can be computed by multiplying the potentials with the atomic charges of the latter. This approach is extensively used in computing pair-wise interaction energies in several pKa's prediction packages, such as MutiConformation Continuum Electrostatics (MCCE) (Alexov and Gunner, 1997, 1999; Georgescu et al., 2002; Song et al., 2009) and DelPhiPKa (Wang et al., 2015, 2016; Pahari et al., 2018).

Once the screened Coulombic interaction energy is computed (via PB or other method), one can deliver the corresponding electrostatic force by taking negative gradient of it. This approach is extensively used in receptor-ligand docking modeling. Recently we developed a tool, the DelPhiForce, which calculates the electrostatic force on a target partner generated by the other partner(source) (Li et al., 2017b,c). In a series of works, it has been demonstrated that the electrostatic force guides binding partners toward their binding positions and orientations. Furthermore, it was pointed out that electrostatic force as a function of the distance between the partners is not monotonic. In case of a microtubule binding domain (MTBD) which binds to microtubule (MT), we demonstrated that the electrostatic force of interaction is attractive when the MTBD is not physically bound to MT, but becomes repulsive when there is a physical contact between MTBD and MT (Li et al., 2016b). Similar effect was found in the case of MTBD interacting with intrinsically disordered E-hooks of MT. This effect was referred as “soft landing” since the electrostatic force de-accelerates the approach of MTBD toward the MT and reduces the landing speed (Tajielyato et al., 2018).

Here we investigated the role of electrostatic forces on molecular recognition using large set of protein-protein complexes with available 3D structures. Particular emphasis is paid on the electrostatic force profile as a function of the distance between the partners. Thus, using the 3D structures of the protein-protein complexes, we moved one of the partners away from the other one in a stepwise manner and at each step the electrostatic forces between them was computed. The electrostatic force as a function of distance between the partners renders an electrostatic force profile. We obtained these profile using two different dielectric distribution models, the traditional 2-dielectric PB protocol assigning low dielectric constant of proteins and high dielectric constant of the water phase, and Gaussian-based smooth dielectric function protocol implemented in DelPhi (Li et al., 2013, 2014, 2015). The goal is to identify common electrostatic force profiles and to use them to infer common roles the electrostatics pays in macromolecular recognitions.

## Methods

### Dataset of Protein-Protein Complexes

The initial set of 603 protein-protein complexes was obtained from a database created by Ray Luo's group at UCI (http://rayl0.bio.uci.edu/rayl) (Berman et al., 2000). We used this set previously to evaluate the parametrical and numerical factors that influence the electrostatic component of binding energy (Chakavorty et al., 2016). A pre-processed dataset was created by selectively extracting dimers (Chakavorty et al., 2016). Modified residues, present in some complexes, were mutated back to their wild type residues as mentioned in their PDB file's header. Furthermore, the proteins with missing terminal residues and duplicated residues were removed. The rest of the protein complexes were then protonated to allow ARG/LYS and GLU/ASP residues to bear a net charge of +1 and −1, respectively. Five hundred steps of steepest decent minimization was performed on the complexes using NAMDv2.9 (Phillips et al., 2005) with Generalized Born implicit solvent (GBIS) model (Onufriev et al., 2000, 2004; Tanner et al., 2011) in the conjunction with CHARMM force filed (MacKerell et al., 1998). The ion concentration was set to zero. The value of 12 Å was selected to calculate the Born radii based on the extent of desired de-screening outlined the Bashford–Case model (Onufriev et al., 2000, 2004; Tanner et al., 2011). Furthermore, the cut-off for non-bounded forces was set to 14 Å and all the other requisite parameters were kept at their default values.

### Finding the Direction of Separation

**Figure 1:**
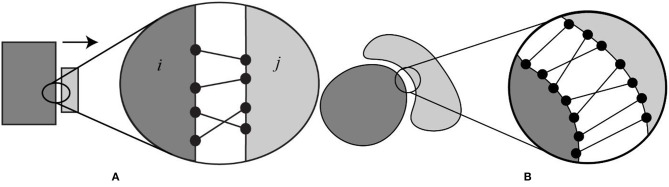
Schematic presentation of the protocol used to separate the binding partners in protein-protein complex. **(A)** Hypothetical case with a flat binding interface and **(B)** case of a binding interface which is not flat. Black dots show the atom pairs across the binding surface that lie within some cut-off distance. Atoms pairs are connected with black lines.

Since the goal of the investigation was to model the role of electrostatics in the bound state and unbound states, we generated a set of configurations of a complex where the partners were separated at various distances. It is understood that the binding process is a complicated event that involves small or large conformational changes. It is also understood that the binding trajectory does not have to be a straight line and that, binding partners may recognize each other via alternative trajectories. However, modeling the conformational changes and different binding trajectories was not the main focus of this work. Instead, we restricted this investigation to cases that do not involve large conformational change and will be assumed that binding partners preserve their conformation in bound and unbound state (called rigid body protocol). In addition, we also assumed that the binding occurs via a single trajectory, which is a straight-line, more or less perpendicular to the binding interface. For the purpose of generating positions for unbound monomers, we developed a protocol to separate bound monomers at various distances along a certain direction of separation (see Figure 1A for schematic representation of the protocol). The first step was to find the direction of separation. Consider a protein complex with a flat binding interface (Figure 1A). We identified all the atom pairs (atom from partner A and atom from partner B) that are within a cut-off distance (5 Å, d_ij_ < 5 Å in Figure 1A). Based on the pair coordinates of the atom “*i*” and “*j*” in each pair, we defined a vector (${\vec{U}}_{ij}$), that connects their centers. The separation direction (vector) of that complex was defined as the average of all the ${\vec{U}}_{ij}$, i.e., the vector sum of atom pairs vectors (${\vec{U}}_{ij}$) divided by the number of atom pairs (*n*). The resultant vector $\vec{A}$ can be expressed as:

(1) $$\text{A }=\left(\frac{\sum {\vec{\text{U}}}_{ij}}{\text{n}}\right)$$

If the binding interface is not flat (Figure 1B), one can apply the above-mentioned approach as well. The outcome depends on the geometry and packing. Another factor to consider is the conformational changes induce by binding. In some cases, the bund molecules could not be separated without introducing artificial overlaps and were deemed unfit for this particular study. Such cases were removed from the dataset.

### Procedures to Remove Cases That Are Not Appropriate for This Study (Pruning the Dataset)

Two protocols were applied to identify such cases. A complex was considered unsuitable if (a) the separation causes atomic overlaps between atoms of partners, (b) the separation resulted in sliding of one of the partners over the other.

#### Removing Cases With Atomic Overlaps

The above protocol was designed to identify pairs of atoms across the binding interface which lie within some cut-off distance. That ideally means that if the protein chains are separated in a correct direction, the distance between atoms in these pairs should only increase after separation (Figure S1A). To check for that, we recomputed these pair-wise distances after the protein chains were separated by 1 Å. If more that 80% of these distances were larger than in bound state, the protein complex was retained in the dataset, otherwise it was removed (examples are shown in Figure S2).

A second pruning was made by considering the partners already separated at 10 Å distance (Figure S1B). In that configuration, we computed the distance between atoms of the partners and if a pair with a distance <4 Å was found, the complex was removed for the dataset. Examples of such cases are provided in Figure S3.

#### Filtering Protein Complexes by Average Distance

**Figure 2:**
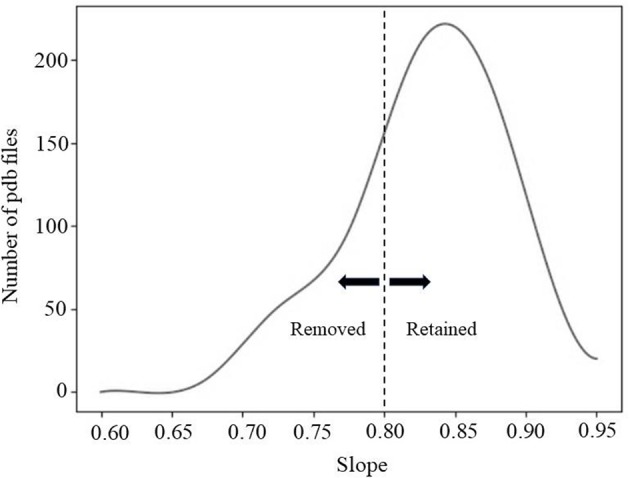
The fitting line slope distribution of 550 protein complexes.

Due to the complex shape of some of the interfaces, the direction of separation may not have been correctly detected by our simple method. Thus, a second screening protocol was applied to remove such cases. It was done by calculating the average distance of atom pairs (known atom pairs found from the method of separation) at each distance of separation. Details of computation are provided in Supplementary Material. Essentially, one expects that if the direction of separation is correctly predicted, the averaged distance between all atom pair should be a linear function of separation distance and the slope of the line should be 1 (Figure S4). Figure 2 shows the distribution of the slope over 550 protein complexes. For the purposes of this study, we removed all cases with slope <0.8. Some examples are provided in Figure S5.

After the pruning protocols, the total number of protein complexes in the dataset was reduced to 275. The list is provided in Supplementary Material.

### Smooth Gaussian-Based Dielectric Function

The energy and electrostatic force were calculated using two different models, which are 2-dielectric model and Gaussian-based dielectric model. In the Gaussian-based dielectric model, the dielectric function is delivered using a representation of atomic densities. Thus, given a macromolecule immersed in a water (solvent), the density of an atom *i* is represented by a Gaussian function (atomic density at position *r* generated by atom *i*) (Chakravorty et al., 2018),

(2) $$\begin{array}{l} \rho_{i}\left(r\right)=\exp\left[-r_{i}^{2}/\left(\sigma^{2}.R_{i}^{2}\right)\right] \end{array}$$

where ρ_*i*_(*r*) is the density at position *r*, *r*_*i*_ is the distance between the center of the atom *i* and position *r*, *R*_*i*_is the van der Waals (vdW) radius of atom *i*, and σ is the variance.

Then, the total atomic density is calculated as follows:

(3) $$\begin{array}{l} \rho_{mol}\left(r\right)=1-\underset{i}{\prod }\left[1-\rho_{i}\left(r\right)\right], \end{array}$$

where ρ_*mol*_(*r*) is the total atomic density at position *r* produced by the whole molecule. According to the Equation (3), the density of the overlapping regions is higher than of each of the single atoms, but never gets above 1 Finally, the smooth dielectric function derived using the atomic density distribution is:

(4) $$\begin{array}{l} \varepsilon \left(r\right)=\rho_{mol}\left(r\right).\epsilon_{in}+\left(1-\rho_{mol}\left(r\right)\right).\epsilon_{out} \end{array}$$

In this equation ε(*r*) is dielectric function for the entire space being modeled, and ϵ_*in*_ and ϵ_*out*_ are the reference dielectric values for the molecule and solvent, respectively.

### Calculating Electrostatics Forces

The goal of this work was to calculate electrostatic forces acting between partners at bound state (physical binding) and unbound distance marked by various separation distances. It was done using DelPhiForce and the corresponding force is termed interaction force (F_int_). The forces were calculated under two different descriptions of the dielectric property of the system (proteins and water phase). In the first approach, termed as the traditional 2-dielectric model, the proteins were considered to be uniform low dielectric cavities immersed in water continuum with high dielectric constant, with a sharp dielectric jump at the protein-water interface. In the second approach, the dielectric properties of the system were modeled via smooth Gaussian-based dielectric function, which ensures that there is a smooth transition of dielectric value between protein and water, including cases in which there is no physical binding (Chakravorty et al., 2018). In addition, the protein themselves feature an inhomogeneous dielectric distribution as opposed to a single dielectric constant [see (Schutz and Warshel, 2001; Song, 2002), for more details].

DelPhiForce delivered electrostatic force has three components which are along the x, y, and z directions. They were used to find the total force and then the total force was projected onto the direction of separation.

Since the modeling is done as a function of the distance between the partners, it is tempting to compute the total electrostatic energy (columbic and polar de-solvation energies) and to take negative gradient to obtain the corresponding forces. Thus, the total electrostatic association energy was computed as the energy difference between molecules being at a particular distance (including distance equal to zero, physically bound state) and free state (unbound molecules). Such an energy difference will be termed electrostatic energy of association, which at physically bound state is the electrostatic binding energy (Equation 5). Once the electrostatic association energies were obtained, we took the gradient to deliver the association force, F_ene_ (Equation 6).

The electrostatic component of association energy for a protein complex (ΔE_*ele*_), with two chains (1 and 2) is given by the difference of the total electrostatic energy of the complex (E_complex_) and of the free molecules (E_1_ and E_2_) as:

(5) $$\begin{array}{l} \Delta {\text{E}}_{\text{ele}}=\;{\text{E}}_{\text{complex}}-\;{\text{E}}_{1}-{\text{E}}_{2} \end{array}$$

Here *E*_*complex*_, *E*_1_and *E*_2_ are the total electrostatic energies of the complex and the individual monomers, respectively (Petukh et al., 2015b). When treated using the 2-dielectric model, the total electrostatic energy of any system was obtained as the sum of the polar solvation energy and the Coulombic energy. When treated using the Gaussian-base dielectric model, the same was delivered by the system's grid energy. All of these energies were computed using DelPhi (Li et al., 2013, 2014). From the electrostatic component of the association energy, we obtained the electrostatic force as:

(6) $$\begin{array}{l} F_{ene}=\;-grad\left(△E_{ele}\right) \end{array}$$

It is anticipated that at large distances, e.g., at distances of 10 Å, F_int_ and F_ene_ will be the same, since at such distances the de-solvation energy is practically zero. However, as the distance between partners decreases, the desolation energy increases and then one should expect that F_int_ and F_ene_ will be different.

## Results

Using the method described above, the chains of protein complexes were separated at distances varying from zero to 10 Å, in steps of 1 Å. Several cases are illustrated in Figure S6. For each complex, we computed the corresponding energies and forces and plotted them as a function of the distance between monomers, resulting in a force profile. Each force profile was analyzed in terms of the following characteristics: (a) at what distance the force along the direction of separation was at its minimum (most attractive force) and (b) does it change its sign as a function of the distance (attractive vs. repulsive). Based on these features, we have outlined the results for F_int_ and F_ene_ separately, as presented below.

### Electrostatic Force of Interaction (F_int_)

**Figure 3:**
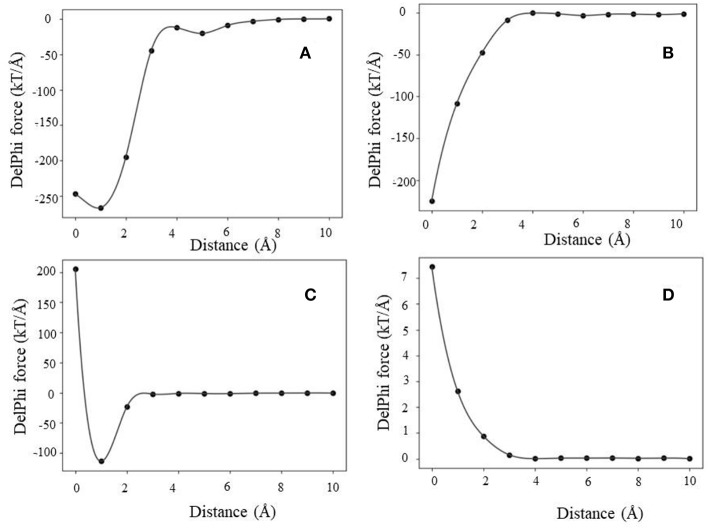
Representative examples of F_int_ profiles obtained using the 2-dielectric model: **(A)** maximum attraction force at a particular distance, **(B)** maximum attraction force at bound state, **(C)** soft landing, and **(D)** repulsive force.

The analysis of the F_int_ (traditional 2-dielectric model) profiles resulted in four distinctive categories. We term them as (a) maximum attraction force at a particular distance; (b) maximum attraction force in the bound state; (c) soft landing and (d) repulsive force. Representative F_int_ profiles are shown in Figure 3.

In case of maximum attraction force at a particular distance (Figure 3A), the force profile is a smooth function with a single minimum, corresponding to negative F_int_ (attractive force). At large distances, the force is small and as the distance decreases, it becomes more negative reaching maximal absolute value at a particular distance (d_1_). Further decrease of the distance until the physical binding (d = 0) makes F_int_ less attractive.

Next is the case of maximum attraction force in the bound state (Figure 3B). The F_int_ profile is a smooth curve attaining maximum absolute value in the bound state (d = 0). In this case, F_int_ provides constant assistance for the binding and the contribution is the largest at the bound state.

The third case is that of soft landing (Figure 3C), where the F_int_ profile has minimum at a particular distance where F_int_ is attractive, but as the distance decreases further, the F_int_ becomes repulsive (positive). Following our previous work, we term those as “soft landing” (Li et al., 2016b; Tajielyato et al., 2018). The reason for that is that F_int_ slows down the approach of the partners toward each other and thus provides soft binding.

Last is the case of a repulsive force (Figure 3D). As it can be seen, the F_int_ profile is a smooth curve, but it is always repulsive (positive). Obviously in such cases, the electrostatics is not the driving force for binding.

**Figure 4:**
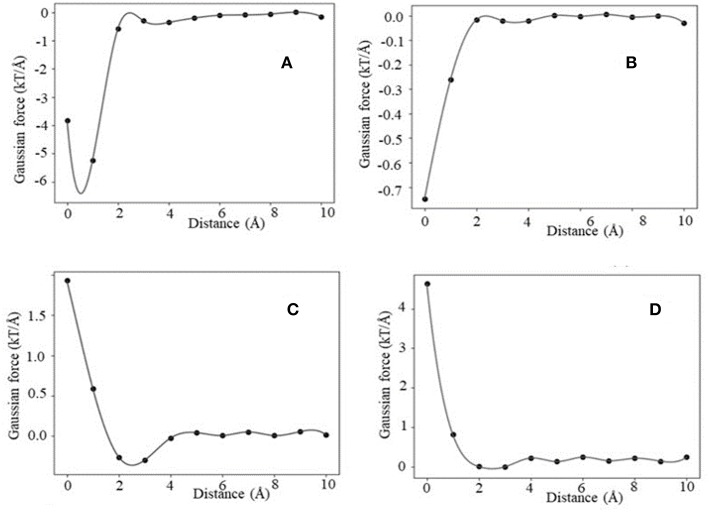
Representative example of F_int_ profiles obtained using the Gaussian-based dielectric model: **(A)** maximum attraction force at a particular distance, **(B)** distance maximum attraction force at bound state, **(C)** soft landing, and **(D)** repulsive force.

Similarly, using Gaussian-based approach we identified the same four distinctive categories of force profiles (F_int_) (Figure 4). However, the magnitude of the corresponding forces is smaller than in case of 2-dielectric model. This is due to larger value of the dielectric function between monomers being at short distance as compared with the 2-dielectric model. Note that these results were obtained with a particular parameter of Gaussian function, namely sigma = 0.96. In our previous work we demonstrated that sigma = 0.93–0.96 is the best for small molecule energy transfer and pKa's of mutants (Li et al., 2013, 2014; Wang et al., 2015), while sigma = 0.7 is the optimal parameter for modeling pKa's of wild type residues (Wang et al., 2015, 2016). If one uses sigma = 0.7, then Gaussian-based DelPhiForce finds only three categories described for traditional 2-dielectric approach (Figure S7). This illustrates the sensitivity of results with respect with sigma value.

### Force Calculated Via Association Energy (F_ene_)

**Figure 5:**
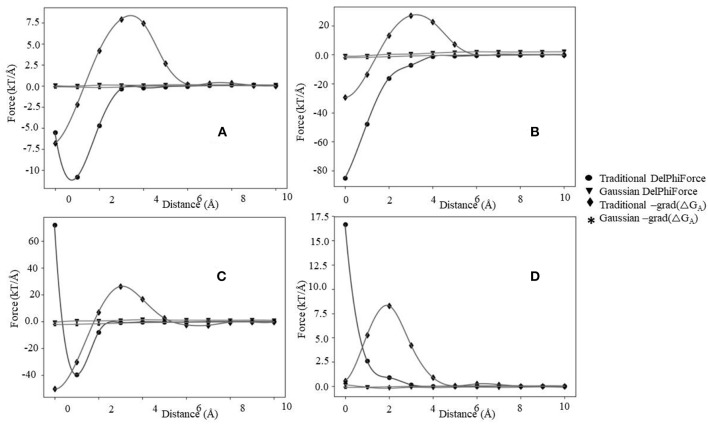
The electrostatic forces (F_int_ and F_ene_) profiles, **(A)** maximum attraction force at a particular distance, **(B)** maximum attraction force in the bound state, **(C)** soft landing, and **(D)** repulsive force.

The association energy includes Coulombic and de-solvation energies, and hence its gradient has two components. The de-solvation energy is expected to vanish at large distances, like at 10 Å, and thus the F_ene_ should be equal to F_int_. In this section, we will use the F_int_ categories described above and will compare them with F_ene_ (Figure 5, Figures S8, S9). Since the magnitude of the forces calculated with Gaussian-based model is much smaller than of those modeled with 2-dielectric approach, the readers are advised to examine Figures S8, S9 prior focusing on Figure 5. The first observation that can be made (Figure 5) is that at large distances both protocols deliver identical or very similar forces (within numerical error). Second observation is Gaussian-based forces are much smaller in magnitude than forces delivered via traditional 2-dielectric model. The magnitude of forces at d = 10 Å is small due to the screening of the water phase and ranges from 0 to 8 kT/Å. As the distance decreases, at about d = 5 Å, one sees that F_int_ and F_ene_ calculated with traditional protocol are different (Figure 5 and Figure S8). The F_ene_ in four representative cases exhibits well-defined “bump,” making the F_ene_ repulsive force. Analysis of the corresponding association energy profiles indicates that this is due to the de-solvation penalty. While the de-solvation penalty is almost zero at d > 5 Å, it becomes more and more pronounced at d < 5 Å, resulting in change of the electrostatic association energy that causes electrostatic repulsion. The smallest effect is seen in case of complexes with the same polarity charges, i.e., the cases where F_int_ is repulsive at large distances (Figure S8D). As the distance decreases further, two additional effects take place: (1) direct Coulombic interactions become stronger because the screening of the water is reduced and (2) de-solvation energy becomes favorable for some complexes made of same polarity partners. As result of increased Coulombic interactions, F_ene_ reverses its trend and becomes attractive again for cases where electrostatics favor the binding (Figures 5A–C). In terms of “soft landing,” the F_ene_, has two repulsive regions: the first one occurs at distances 2–4 Å (Figure S8C), and the second at the binding position(d = 0 Å).

Interesting phenomena is observed for cases where the electrostatics disfavor the binding (Figure 4D). While the Coulombic interactions at short distances are repulsive, due to a favorable change of solvation energy, the F_ene_ becomes slightly attractive (Figure 5D). Such favorable “de-solvation” effect was previously described in Bertonati et al. (2007). It is attributed to stronger interactions with water when the same polarity partners form a tight complex compared with their interactions with water in unbound state.

Turning our attention to forces computed with Gaussian-based smooth dielectric function, one can see that F_ene_ profile is much smoother than of F_ene_ calculated with traditional 2-dielectric model (Figures S9, S10). There is no “bump” at d = 2–4A, which was observed in case of traditional 2-dielectric model (except for case shown in Figure S9A). The reason is that the dielectric function between monomers in case of Gaussian-based model does not exhibit a sharp jump when the distance between interfaces is about a size of a water molecule [this was discussed in details in our previous work (Chakravorty et al., 2018)]. One observes that the profiles are quire dependent on the choice of sigma. If sigma = 0.7, the force profiles of F_int_ and F_ene_ are similar (Figure S10), while if sigma = 0.96 (Figure S9), they are quite different. The main difference is that at sigma = 0.96 the F_ene_ is calculated to be positive (repulsive) at bound position. Large sigma effectively means that the size of macromolecules increases and thus their binding interface increases as well, resulting in larger de-solvation penalty compared with cases with small sigma. This is the reason for F_ene_ to be positive at bound state if sigma = 0.96. It should be clarified that this observation should be considered with caution, since it depends on the dataset. If such an investigation, for example is done for a protein complex that is electrostatically driven, like barnase-barstar, the calculated F_ene_ via the Gaussian-based dielectric model is attractive for all distances.

### Electrostatic Profiles Types and Corresponding Charges

In this paragraph, we present our finding about the relationship of the four categories force profiles (based on traditional 2-dielectric model to calculate F_int_) with the polarity of the net charge of the monomers and their interfaces. Table 1 provides information about the total number of cases in each category and the number of cases with same polarity, different polarity and zero charge of the partners, in terms of the net charge and the charge of the corresponding interfaces. One can see that the vast majority of the cases corresponds to forces being maximal in the bound state, while the other categories are less represented. There appears to be a tendency that cases for which the force is repulsive are also the cases made of monomers with the same polarity of charge, which is overcompensated by the large fraction of cases having opposite polarity of the interface.

**Table 1:** The percentage of the polarity of net and interfacial charges of partners for each protein complexes.

**Categories**	**# PDB files**	**Same polarity on the partners (%)**		**Zero charge on one of the partners (%)**		**Opposite Polarity on the partners (%)**	
		**Total charge**	**Binding surface**	**Total charge**	**Binding surface**	**Total charge**	**Binding surface**
Max_F_at bound position	204	35.0	30.70	20	7.92	45.0	61.39
Max_F at particular distance	16	30.0	35.00	25	25.00	45.0	40.00
Soft landing	35	40.0	37.14	23	8.97	37.0	54.29
Repulsive	20	46.5	12.50	20	25.00	33.5	62.50

## Conclusion and Discussion

This work investigated the role of electrostatic force in molecular recognition, and we would like to emphasize that the focus is on recognition, rather on binding. Thus, the role was evaluated along a plausible trajectory, not just at bound state. Thus, while there are quite many papers dealing with the role of electrostatic in macromolecular binding, they typically address the question “does electrostatics facilitates the binding?” (Brock et al., 2007; Talley et al., 2008; Chen et al., 2012; Munde et al., 2013; Sundlass et al., 2013; Chakavorty et al., 2016; Ghaemi et al., 2017). Here we turned our attention to electrostatic force contribution to the macromolecular recognition by computing electrostatic force along plausible binding trajectory via numerous protocols. The goal was to investigate if electrostatic force as a function of separation distance follows Coulomb law and its magnitude is inversely proportional to the square of distance. Two electrostatic forces were modeled: electrostatic force of interactions (F_int_) and the electrostatic force delivered as negative gradient of electrostatic association energy (F_ene_).

It should be said that F_int_ and F_ene_ are different and reflect different aspects of the association process. The F_int_ infers how the receptor attracts/repels the ligand along the ligand trajectory toward the binding pocket of the receptor. The F_ene_ indicates how the total electrostatics of the combined system made of receptor, ligand and solvent affects the ligand approach toward the receptor.

The results indicate that if one applies the traditional 2-dielectric or Gaussian-based protocol with large sigma, four scenarios (four force profiles) can be identified for F_int_. Two of them, namely profiles termed “maximum force at bound state” and “repulsive force” in general follow the standard Coulomb inverse square distance formula. However, the other two, namely “maximum force at a particular distance” and “soft landing” have much more complicated distance dependence. This is especially noticeable for “soft landing” case, where the force reverses its direction as the distance decreases, from being attractive to being repulsive at short distances. If one takes the F_ene_ on the same representative complexes, the resulting profiles are dramatically different at short separation distances. In all cases considered in this study, the F_ene_ calculated with traditional 2-dielectric protocol has a positive bump (peak of repulsive force) at distance of about 2–4 A (the average size of a water molecule). This is due to the traditional 2-dielectric protocol, such that if the water molecule cannot geometrically propagate in-between the separated monomers, then the space is filled with low dielectric media (with dielectric constant of solute) and this results in large increase of the de-solvation penalty. This is the reason for the positive peak of the F_ene_ at short distances. Even more, because of this sharp increase of de-solvation penalty, one no longer observes the case of “soft landing,” since the negative (attractive) peak of force seen in F_int_ profile at a particular distance is now overwhelmed by the large de-solvation penalty.

Turning our attention to results for F_int_ obtained with Gaussian-based smooth dielectric function with small sigma, one sees that in our dataset we cannot detect a case that can be classified as “soft landing.” It should be recalled (Table 1) that “soft landing” cases outlined above for the traditional 2-dielectric model and Gaussian model with large sigma were representing complexes mostly made of monomers with same polarity charge but having complementary polarity on interfaces. Furthermore, at short distances the favorable electrostatic interactions across the interface in the traditional 2-dielctric protocol and Gaussian protocol with large sigma are very strong due to the low dielectric between interfaces (water is not able to penetrate in-between the monomers). In contrast, in Gaussian-based protocol with small sigma, the space in-between the monomers even situated at short distances is still occupied by relatively high dielectric constant (Chakravorty et al., 2018), which reduces the favorable interactions and thus does not allow for force minima.

Switching to F_ene_, calculated with Gaussian-based protocol using sigma = 0.7, one sees that only two cases can be identified: “maximal force at bond state” and “maximal force at a particular distance.” Similarly, to traditional 2-dielectric protocol, “soft landing” case is not observed, while the Gaussian-based protocol of computing F_ene_ does not indicate a case of “repulsing” force. Even more, the gaussian-based protocol with sigma = 0.96 predicts that the corresponding F_ene_ are always repulsive.

While the force profiles calculated with Gaussian-based protocol depend on selection of sigma and their magnitude is smaller than those calculated with traditional 2-dielectric model, still the main message is that the force profiles are quire irregular. It is understood that this statement is based on the assumption that the ligand approaches the receptor via straight trajectory perpendicular to the binding interface, and in many cases such a single trajectory may not be reflecting actual recognition process. However, we simply want to demonstrate that electrostatic forces contribution to the recognition is very complex and their magnitude and direction may change as ligand approaches the receptor. Taking into consideration all other forces contributing to the binding, including vdW forces, it can be generalized that the binding process involves complex interplay of forces so to assure that physical docking does not result in large strain across the interface.

## Data Availability Statement

The raw data supporting the conclusions of this manuscript will be made available by the authors, without undue reservation, to any qualified researcher.

## Author Contributions

HS carried the research and wrote the manuscript. AC carried the research. EA supervised the research and wrote the manuscript.

### Conflict of Interest

The authors declare that the research was conducted in the absence of any commercial or financial relationships that could be construed as a potential conflict of interest.
