# Supplementary material for: Modeling Electrostatic Force in Protein-Protein Recognition
Source: Front Mol Biosci. 2019 Sep 25;6:94. doi: 10.3389/fmolb.2019.00094 (PMC6774301; doi:10.3389/fmolb.2019.00094)
Supplement: Supplementary file 1 [file Table_1.docx]

Supplementary Data for

Modeling electrostatic force in protein-protein recognition

H. B. Mihiri Shashikala, Arghya Chakravorty, and Emil Alexov

*Removing cases with atomic overlaps:*


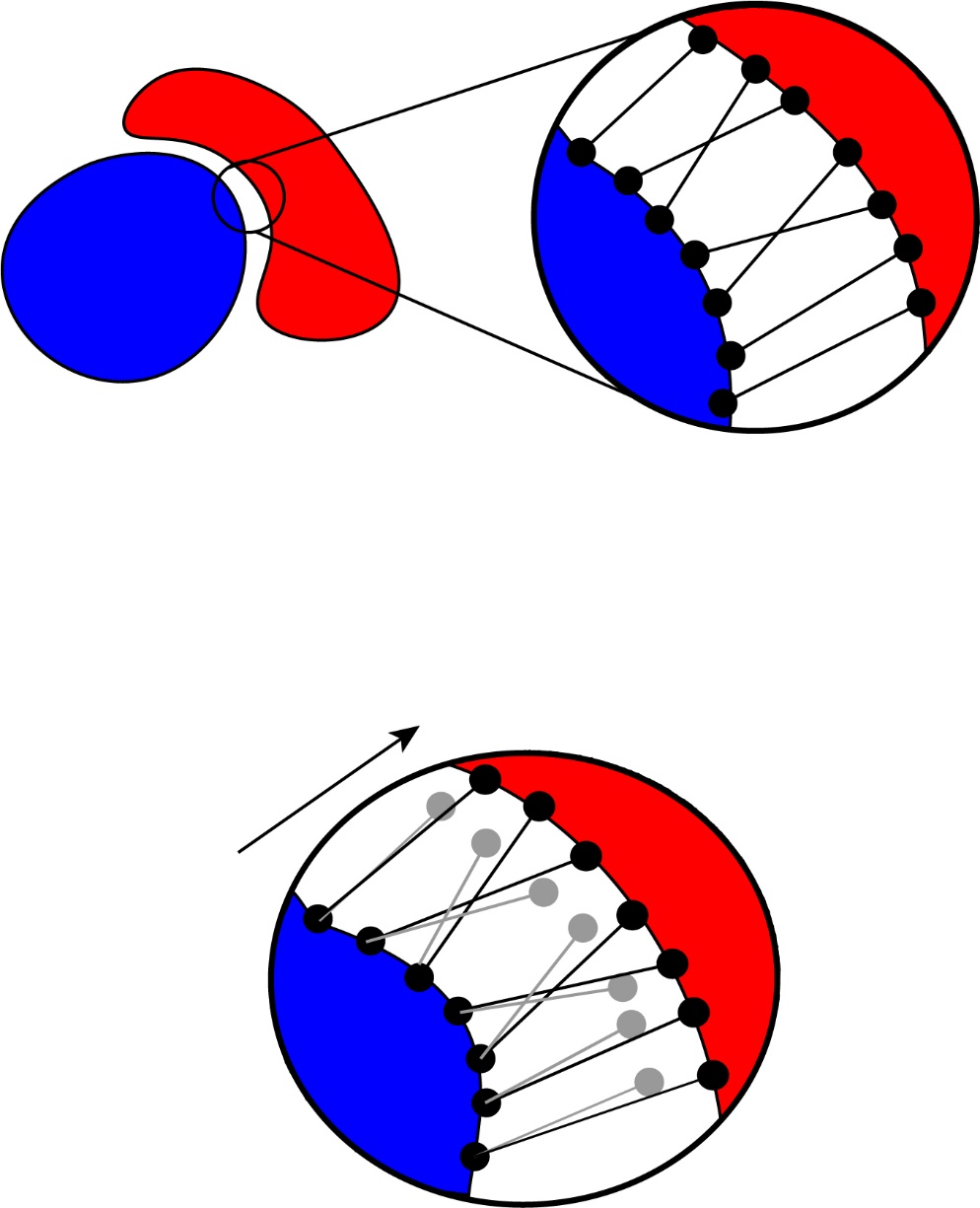

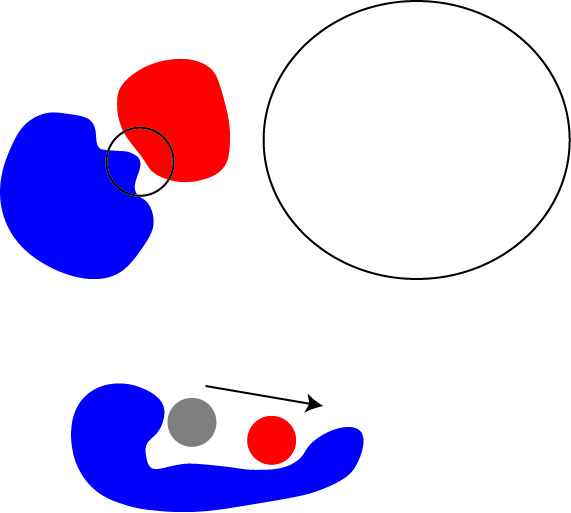


1. (b)

**Figure S1.** The schematic diagram for a protein complex. (a) Gray and black lines show the atom pairs in protein complex at bound state and after separating monomer by 1Å distance. The separated bound monomer direction is shown by bold arrow. (b) schematic representation of a case of protein complex in which the separated monomer slides over its partner (this is an indication of a wrong direction of separation).


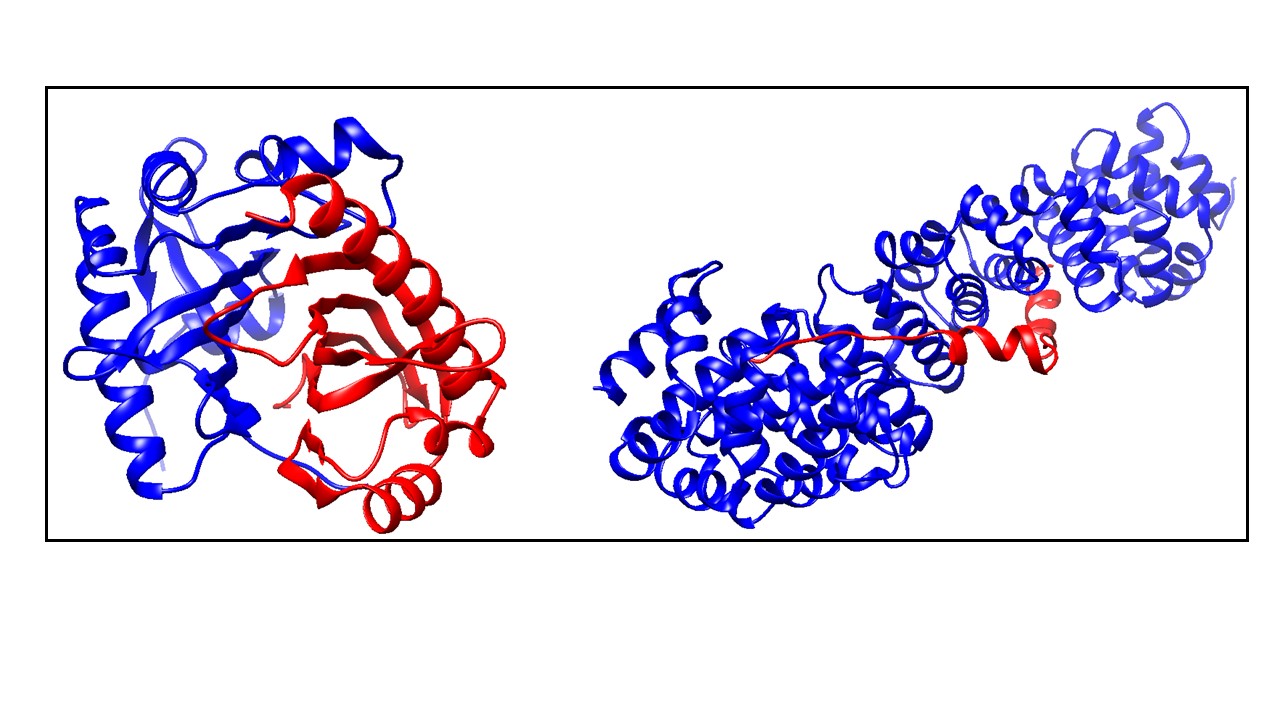


**Figure S2.** The removed protein complex from the dataset, considering the evolution of distance of selected atom pairs.


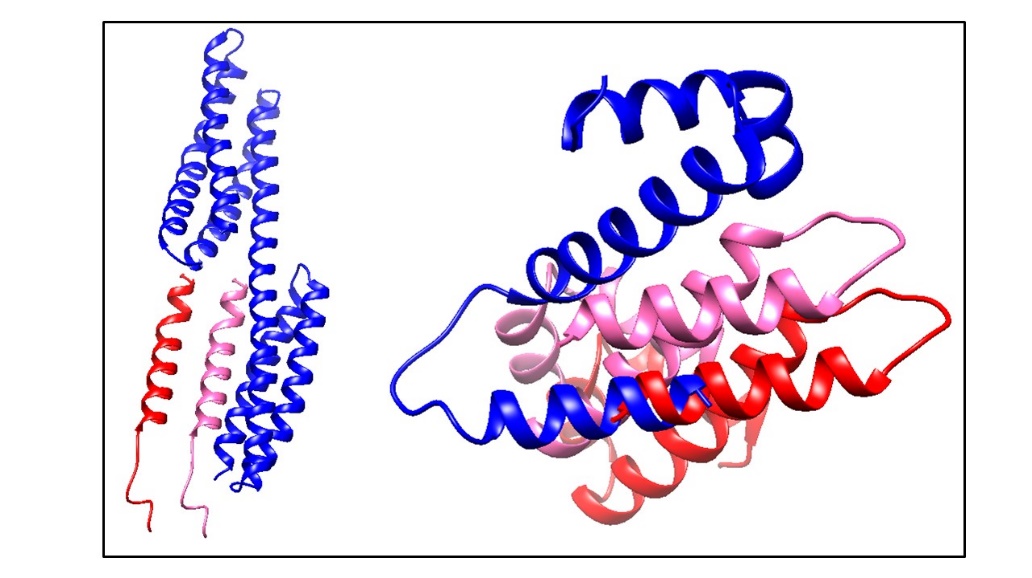


**Figure S3.** The removed protein complexes from the dataset, considering sliding. Blue and pink are the chain 1 and 2 respectively in protein complex and chain 1 is the fixed. Chain 2 is separating based on to our method. Pink and red color show protein at bound and separated by 10 Å respectively.

*Filtering protein complexes by average distance:*

The second procedure were introduced to filtering unwanted protein complexes considering the average distance of known atom pairs

In the average distance method, the slope of the average distance vs separation graph was determined. It was done by calculating average distance of atom pairs (known atom pairs found from method of separation) in each separation for given protein complex. The average distance was calculated as follows:

$$D_{k}=\frac{\sum\left| \vec{U_{ij}} \right|}{n}$$

Where, $D_{k}$ is the average distance and $\vec{U_{ij}}$ is distance in atom pair.


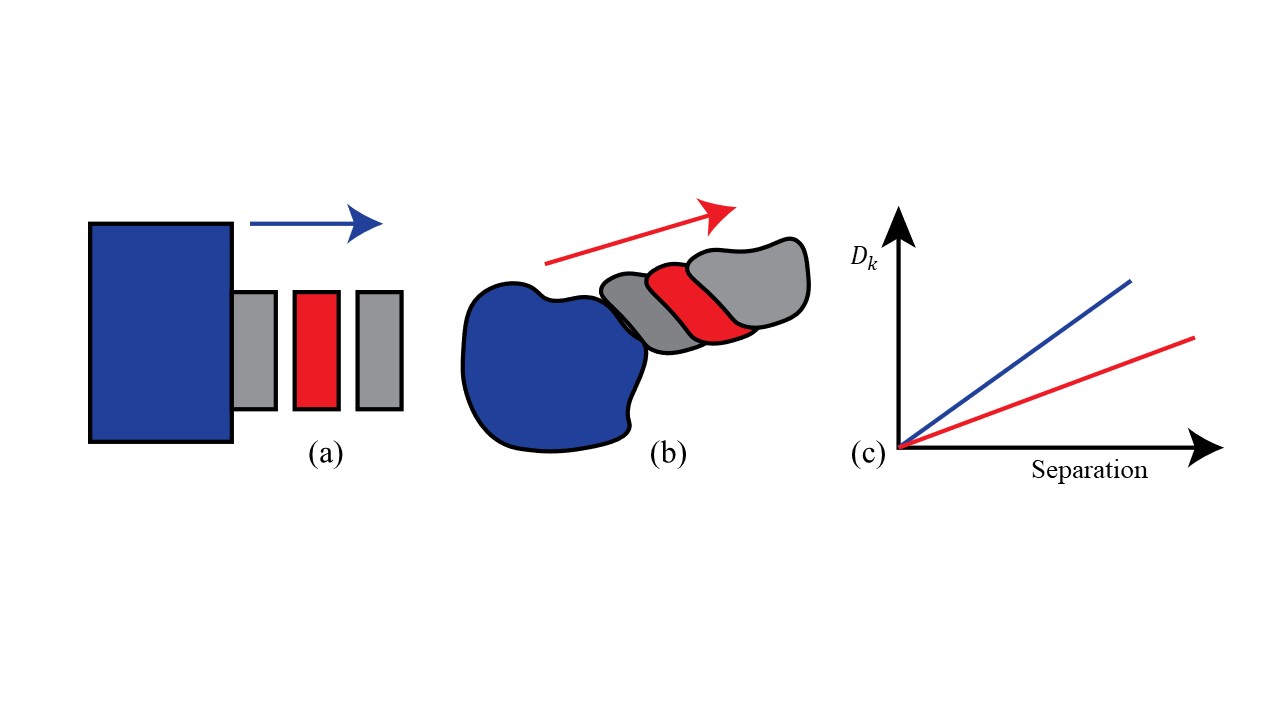


**Figure S4.** The schematic diagram of two protein complexes, separated at various distances ((a) and (b)), and separation direction is representing in arrows. (c) The average distance vs separation for the above two cases ((a) and (b)) shown in blue and red lines, respectively.

For the protein complex with a flat binding surface, the average distance is proportional to the separated distance and the slope of the fitting line is 1. In other cases, the slope may be different from 1 due to complex proteins binding surface (Fig. 5). In figure 6 shows the four different protein complexes with their slopes.


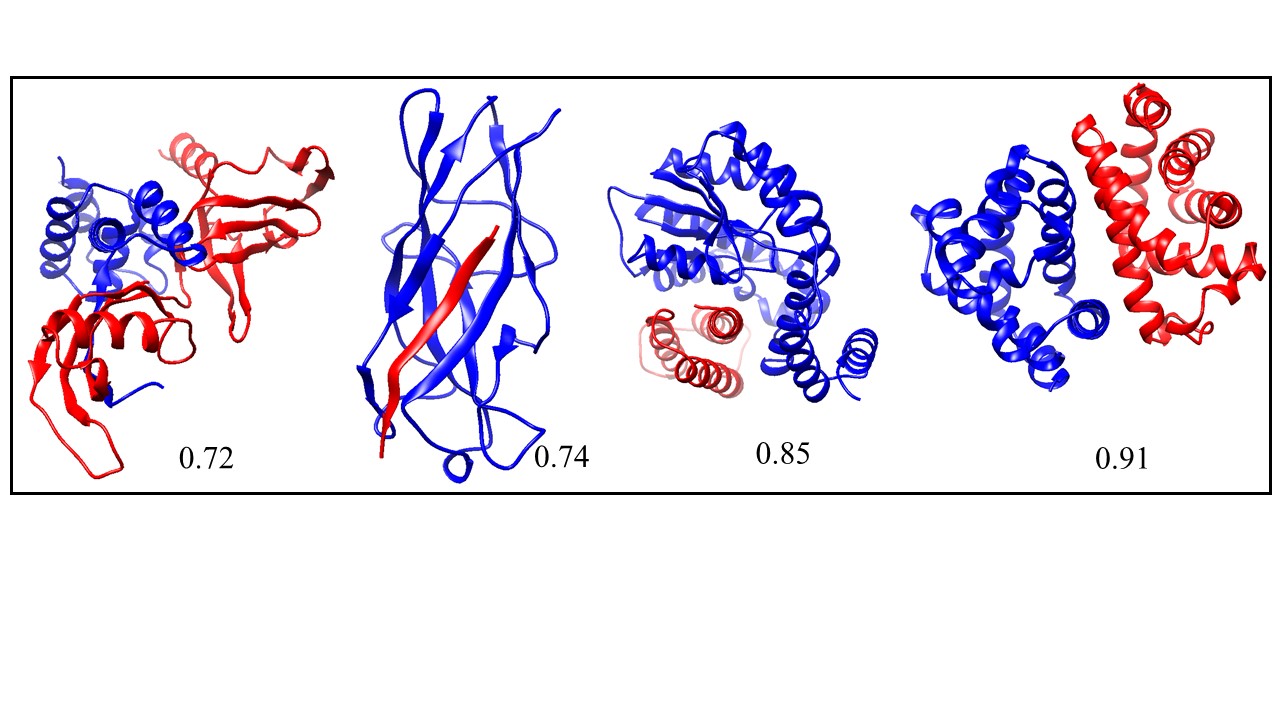


**Figure S5.** The protein complexes at its bond state, including the slope of the fitting line.

List of protein complexes retained in the study after applying purging protocols:

1A9X 1AQW 1BBZ 1BLX 1C1Y 1CG5 1CKA 1CZQ 1CZY 1D3B 1DDV 1DGW 1DTD 1E6Y 1EER 1EEX 1EG4 1ELW 1EVH 1FLT 1FS1 1G6G 1G73 1GCV 1GL2 1GL4 1GVN 1GYB 1H2S 1H9O 1I7Q 1J2X 1JAT 1JEK 1JMX 1JW6 1JYO 1K8K 1KSH 1KYF 1L6X 1LQV 1LSH 1LUC 1MFG 1MIZ 1MZW 1N7F 1NKZ 1NQ7 1NRJ 1OAI 1OAO 1OAQ 1OBX 1OEY 1OO0 1OU8 1OV3 1PBY 1PK1 1PK6 1Q1A 1Q40 1QAV 1QOP 1R0R 1R1Q 1R4P 1REW 1RM6 1S5D 1SCT 1SE0 1SEM 1SHA 1SR4 1SSH 1SVF 1T0P 1T6O 1TA3 1U0S 1U7B 1U8T 1UGH 1UGX 1UJ0 1UPT 1UTI 1V74 1VLF 1W70 1W9E 1WDD 1WMH 1WVE 1WXC 1XG2 1YAR 1YFN 1YMT 1YPH 1YRO 1YTV 1YUC 1YWO 1Z0J 1Z0K 1Z3E 1Z5Y 1Z9O 1ZAV 1ZUK 1ZV8 2A3I 2A5T 2A9K 2AIR 2AKA 2APO 2AQ2 2AQ9 2ARP 2ASU 2B1X 2B3G 2B9H 2BBK 2BCG 2BCN 2BEQ 2BEZ 2BKY 2BMO 2BPT 2BZ8 2CIO 2CWG 2CZV 2D1X 2D7C 2DE6 2DF6 2DJF 2DRM 2DS8 2DZE 2E4M 2F4M 2FCW 2FF4 2FGR 2FLU 2FOJ 2FYM 2G30 2G5L 2GBW 2GH0 2GHT 2GIA 2GPH 2GPO 2H7Z 2HEY 2HO2 2HQH 2HQS 2HT9 2HUE 2HY5 2IG0 2IUH 2IZX 2J12 2J6F 2J7P 2J7Y 2J9U 2JGB 2JJS 2JK9 2NL9 2NNU 2O4J 2O4X 2O9V 2OBH 2ODE 2OVH 2OZN 2P1T 2P45 2P54 2P58 2PU9 2QIY 2QKH 2QWO 2R2 2RI7 2RKY 2RMC 2UWJ 2UYZ 2V1T 2V2F 2V52 2V8C 2V9T 2VLQ 2VN6 2VNF 2VOL 2VPB 2VWF 2VZG 2W0P 2W3O 2WJN 2YVJ 2Z3Q 2Z5B 2Z8P 2ZA4 2ZMI 2ZON 2ZSI 2ZZD 3BC1 3BEJ 3BOM 3BP6 3BRL 3BS5 3BWU 3C6W 3CJS 3CPT 3D1K 3D3B 3D44 3D9T 3DAC 3DDC 3DLQ 3E1R 3EHU 3EJ9 3EJB 3EMW 3ET3 3F1P 3F4Y 3F6Q 3FAP 3FHV 3FJU 3FP2 3FPN 3GJ3 3GL6 3H11 3H6P 3H7H 3H8K 3HDS 3HHS 3HXI 3KB3 3KDJ 3KNB 4UBP 6RLX


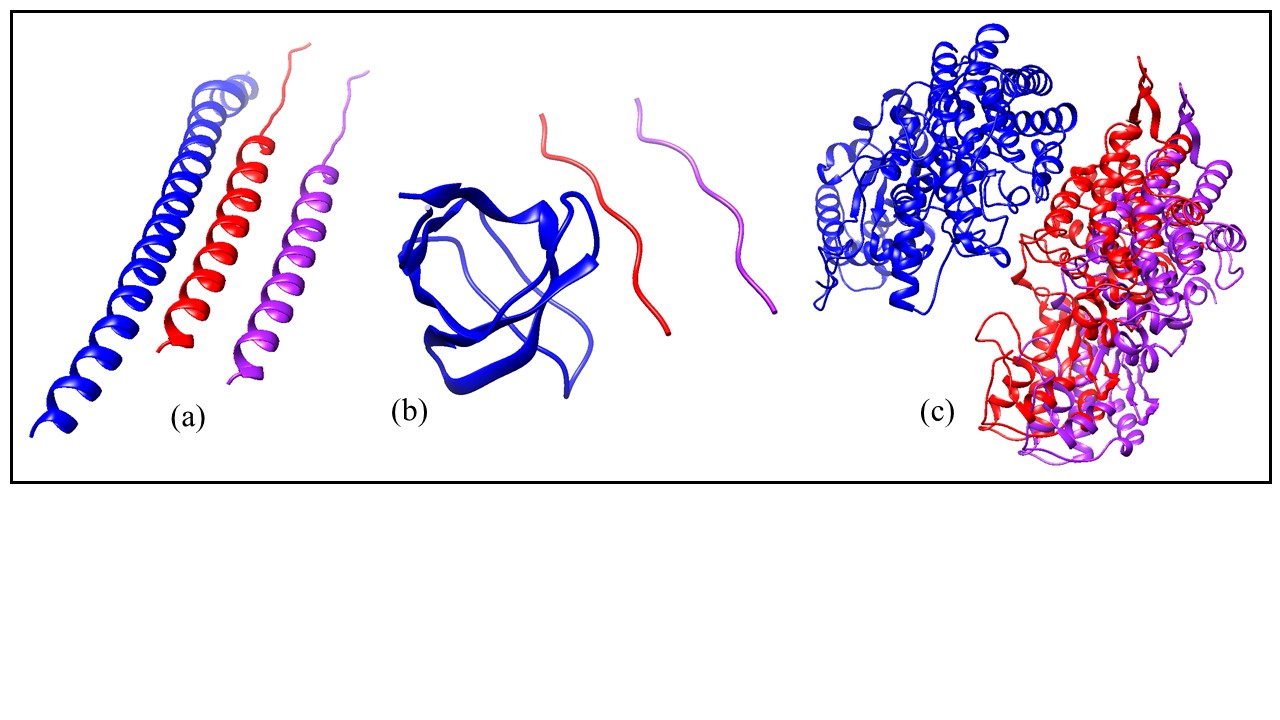


**Figure S6.** Example of three proteins complexes at bound state and being separated by 10 Å. Blue-red, protein complex at bound state. Blue-purple, after separating by 10Å.


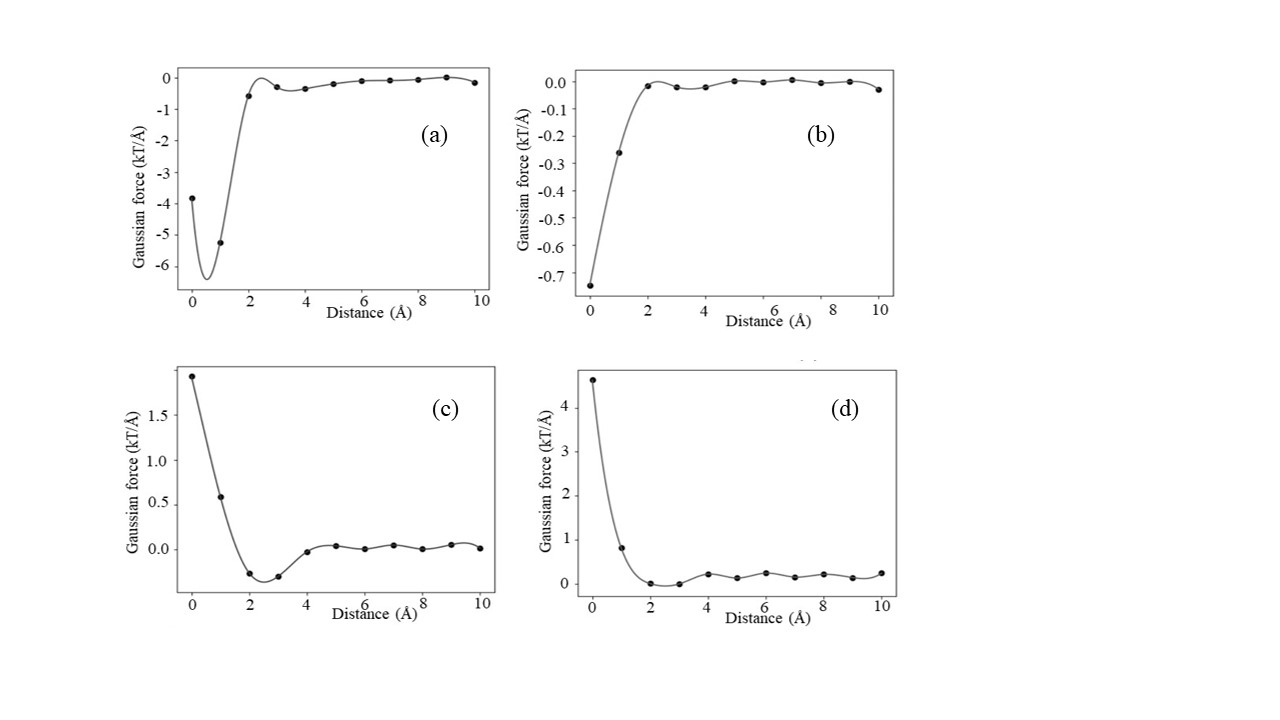


**Figure S7.** The graph for Gaussian-based F_int_ profiles (σ =0.96): (a) maximum attraction force at a particular distance (b) maximum attraction force at bound state (c) soft landing and (d) repulsive force.


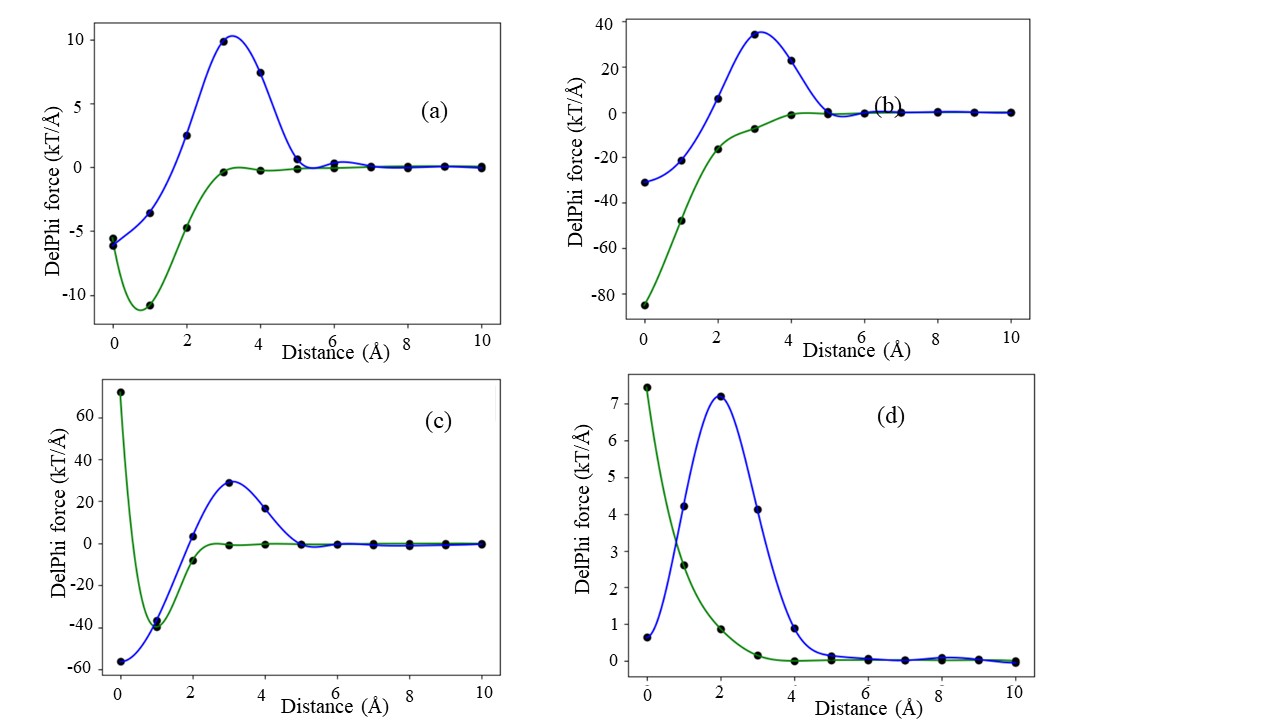


**Figure S8.** Representative of F_int_ and F_ene_ profiles (traditional) are shown in green and blue line respectively: (a) maximum attraction force at a particular distance (b) maximum attraction force at bound state (c) soft landing (d) repulsive force (classification based on F_int_).


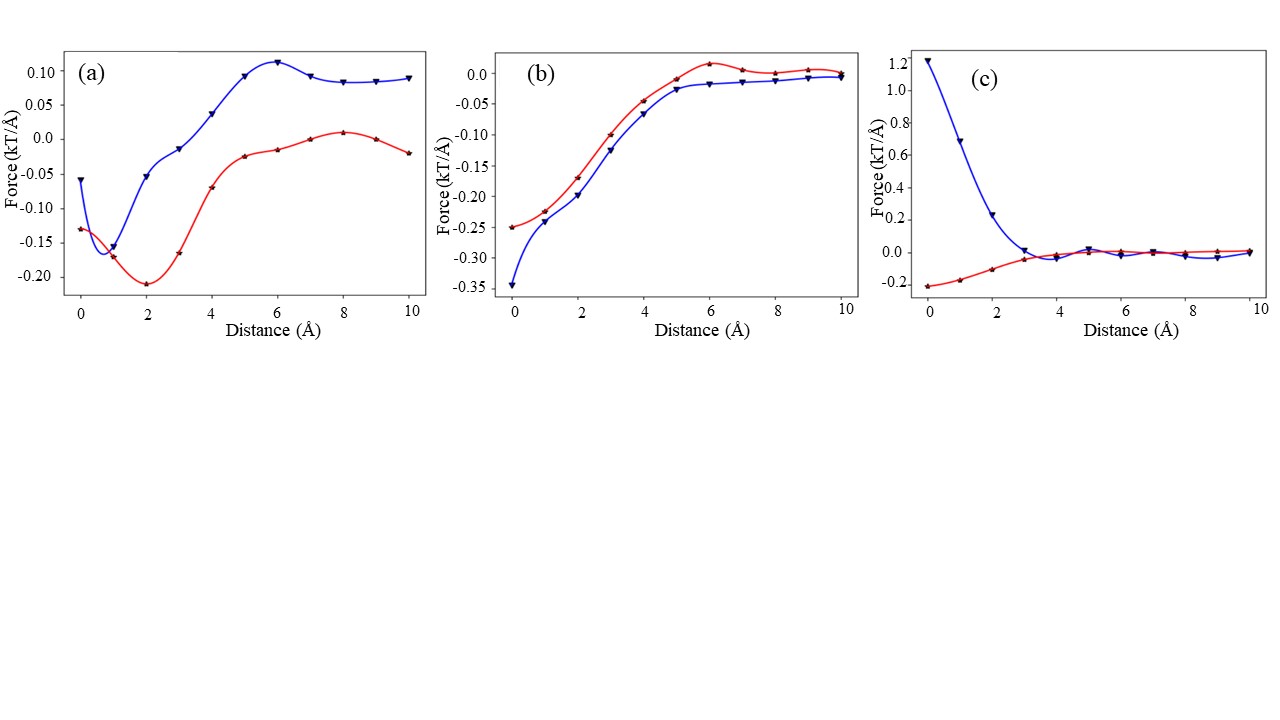


**Figure S9.** Representative of Gaussian(σ=0.70) F_int_ and F_ene_ profiles are shown in blue and red line respectively: (a) maximum attraction force at a particular distance (b) maximum attraction force at bound state (c) repulsive force (classification based on Gaussian-based F_int_).


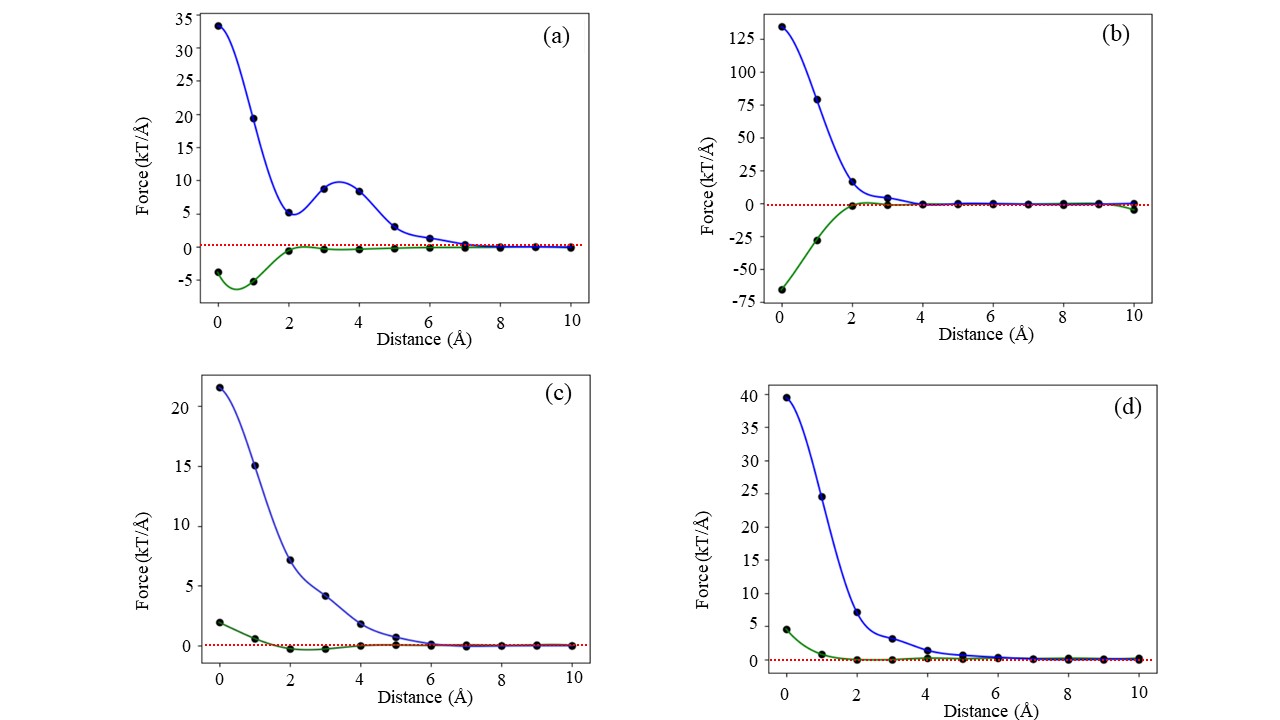


**Figure S10.** Representative of Gaussian(σ=0.96) F_int_ and F_ene_ profiles are shown in green and blue line respectively: (a) maximum attraction force at a particular distance (b) maximum attraction force at bound state (c) soft landing (d) repulsive force (classification based on Gaussian model F_int_). The red dash lines show the zero line to guide the eye.
